# The Role of Awareness on Motor-Sensory Temporal Recalibration

**DOI:** 10.3389/fnint.2022.747544

**Published:** 2022-02-15

**Authors:** Mikaela Bubna, Melanie Y. Lam, Erin K. Cressman

**Affiliations:** ^1^Sensorimotor Control Laboratory, Faculty of Health Sciences, School of Human Kinetics, University of Ottawa, Ottawa, ON, Canada; ^2^Perceptual-Motor Behaviour Laboratory, Department of Human Kinetics, Faculty of Science, St. Francis Xavier University, Antigonish, NS, Canada

**Keywords:** awareness, motor-sensory task, temporal recalibration, temporal order judgment, simultaneity judgment, single tap, repetitive tap, point of subjective simultaneity

## Abstract

Temporal recalibration (TR) may arise to realign asynchronous stimuli after exposure to a short, constant delay between voluntary movement and sensory stimulus. The objective of this study was to determine if awareness of the temporal lag between a motor response (i.e., a keypress) and a sensory event (i.e., a visual flash) is necessary for TR to occur. We further investigated whether manipulating the required motor and perceptual judgment tasks modified the influence of awareness on TR. Participants (*n* = 48) were randomly divided between two groups (Group 1: Aware and Group 2: Unaware). The Aware group was told of the temporal lag between their keypress and visual flash at the beginning of the experiment, whereas the Unaware group was not. All participants completed eight blocks of trials, in which the motor task (single or repetitive tap), perceptual judgment task (judging the temporal order of the keypress in relation to the visual flash or judging whether the two stimuli were simultaneous or not), and fixed temporal lag between keypress and visual flash (0 or 100 ms) varied. TR was determined by comparing judgments between corresponding blocks of trials in which the temporal lag was 0 or 100 ms. Results revealed that both the Aware and Unaware groups demonstrated a similar magnitude of TR across all motor and perceptual judgment tasks, such that the magnitude of TR did not vary between Aware and Unaware participants. These results suggest that awareness of a temporal lag does not influence the magnitude of TR achieved and that motor and perceptual judgment task demands do not modulate the influence of awareness on TR.

## Introduction

In many everyday experiences, we perceive sensory events as arising as a consequence of our movements. For example, when typing on a computer, we expect letters to appear on the screen immediately after pressing a key. The temporal relationship between our action (i.e., pressing a key) and resulting sensory feedback (i.e., a character appearing on the screen) allows us to form a coherent representation of the temporal order of events in the world. Interestingly, it has been shown that the perceived temporal order between events can be disrupted by systematically manipulating the timing between them. In particular, researchers have shown that by introducing a constant (i.e., fixed) temporal lag between an action (keypress) and a subsequent sensory stimulus (a visual flash appearing), one’s perception of the relative timing of events changes. For example, if participants complete several keypresses, after which there is a constant temporal lag between their keypress and a flash appearing on the screen, they begin to associate the lagged sensory feedback (i.e., the flash appearing on the screen) as arising due to their action (i.e., the keypress) (Vroomen et al., 2004; Stetson et al., 2006; Sugano et al., 2010; Stekelenburg et al., 2011; Timm et al., 2014; Machulla et al., 2016). Moreover, if the flash is to suddenly appear immediately after their keypress, participants now perceive the flash as occurring before their keypress. It is assumed that this temporal recalibration (TR) arises to minimise temporal discrepancies between one’s actions and sensory stimuli, enabling one to still infer causality (Stetson et al., 2006).

Temporal recalibration has been shown to arise across paradigms employing various motor tasks (e.g., single tap and repetitive tap tasks; Stetson et al., 2006; Tsujita and Ichikawa, 2016), as well as perceptual judgment tasks [e.g., temporal order judgment (TOJ) and simultaneity judgment (SJ); Vroomen et al., 2004; Vatakis et al., 2008; Love et al., 2013; Machulla et al., 2016]. In a single tap task, participants typically respond as fast as possible to a go signal (e.g., a cross appearing) by pressing a key (Stetson et al., 2006; Timm et al., 2014). In contrast, in a repetitive tap task, participants learn to maintain a consistent keypress tapping pace while a flash is presented at a constant lag following each tap (see Heron et al., 2009; Sugano et al., 2010; Stekelenburg et al., 2011; Keetels and Vroomen, 2012; Yarrow et al., 2013; Tsujita and Ichikawa, 2016). In both tasks, participants are then asked to judge the timing of a flash relative to their keypress, where the flash can appear before or after their keypress (Sugano et al., 2010). Perceptual judgment tasks used to establish TR include asking individuals to either judge the relative timing of two events by indicating which event (e.g., keypress or flash) occurred first (TOJ; see Stetson et al., 2006) or indicate if the two events occurred at the same time (SJ) (see Keetels and Vroomen, 2012). TR is then established if the point of subjective simultaneity (PSS) differs between blocks of trials with varying constant lags (e.g., 0 ms fixed lag vs. 100 ms lag; Stetson et al., 2006). In a TOJ task, the PSS is an indirect measure of simultaneity, corresponding to the stimulus onset asynchrony (SOA) at which participants indicate the keypress (and hence flash) came first 50% of the time (García-Pérez and Alcalá-Quintana, 2015; Kostaski and Vatakis, 2018). In contrast, in the SJ task, the PSS is a direct measure of simultaneity, and is computed by establishing the peak of the psychometric function fitted to the proportion of synchronous responses as a function of SOA (Kostaski and Vatakis, 2018).

While the phenomenon of TR is well documented, the mechanism(s) underlying TR are unclear. Recently, Tsujita and Ichikawa (2016) suggested that awareness of the motor-sensory delay is necessary for TR to arise. In their paradigm, awareness of the motor-sensory delay was modulated directly within a repetitive tapping task in which a flash was presented at a fixed lag of 200 ms following four initial taps. The timing of the flash relative to a final sixth tap varied across trials and participants judged whether the flash occurred before or after their final keypress (i.e., completed a TOJ task). Participants were divided into two groups, an Aware group and an Unaware group. The participants in the Aware group were introduced to the 200 ms flash lag abruptly at the start of testing trials and were informed of the fixed lag. The participants in the Unaware group were introduced to the 200 ms flash lag gradually over the test trials and they were *not* informed of the fixed lag. Results indicated that the Aware group showed significant TR (TR = ∼99 ms), while the Unaware group did not demonstrate significant TR (TR = ∼11 ms). These initial findings support the proposal that awareness of the temporal lag between one’s motor actions and resulting sensory stimuli is necessary for significant TR to arise, at least in a repetitive tap task when a TOJ is required. That said, task demands (i.e., requirements of motor and perceptual judgment tasks) may modulate the influence of awareness on TR.

In this study, we sought to establish the role of awareness in a motor-sensory TR paradigm, where awareness was defined as in Tsujita and Ichikawa (2016). Specifically, awareness was defined as knowledge that a visual flash would appear after a slight delay following one’s keypress. We further looked to determine if the influence of awareness is modulated by motor task and perceptual judgment task demands. Two groups of participants (Group 1: Aware and Group 2: Unaware), completed 8 blocks of test trials in which the motor task (single vs. repetitive tap), perceptual judgment task (TOJ vs. SJ), and fixed temporal lag between keypress and visual flash (e.g., a 0 or 100 ms lag) were varied. In accordance with the proposal put forth by Tsujita and Ichikawa (2016), we hypothesised that the magnitude of TR would be larger in the Aware group compared to the Unaware group. We further hypothesised that TR would be greater when the Aware group performed the repetitive tap task compared to the single tap task as research has demonstrated that rhythmic, repetitive tapping involves cognitive engagement, including working memory (Holm et al., 2017; Meijer and Krampe, 2018), and hence participants may benefit from awareness. We also postulated that TR would be greater in the Aware group completing the TOJ task compared to the SJ task, as behavioural and neurophysiological evidence suggests that the TOJ task is more cognitively demanding than the SJ task (Love et al., 2013; Binder, 2015; Basharat et al., 2018). More specifically, participants have indicated that the TOJ task is more difficult to complete than the SJ task (Love et al., 2013), and cortical areas associated with cognitive processing (e.g., prefrontal cortex, occipito-temporal regions, and superior and inferior parietal lobules) have shown increased recruitment in the TOJ task vs. the SJ task (Binder, 2015). Support for our hypotheses would suggest that awareness drives TR and that task demands modulate the influence of awareness on TR.

## Materials and Methods

### Participants

Participant recruitment and data collection commenced after the Faculty of Health Sciences at the University of Ottawa approved a Safe Research plan and ethical approval was received from the University of Ottawa’s Health Sciences and Science Research Ethics Board. Fifty-two participants (*M* = 24.1 years; SD = 3.9 years; 27 females) were tested, and they all provided written informed consent. Initial sample size taking into account all comparisons (Group, Fixed Lag, Motor Task, and Perceptual Judgement) was determined to be 48 participants by performing a power analysis using G*Power (Version 3.1.9.3; Faul et al., 2007), with a desired power of 0.80, a probability of Type 1 error of 0.05, and an expected effect size (Cohen’s *f* value) of 0.14 based on an estimated mean TR of 88 ms and standard deviation of 63 ms from data provided in Tsujita and Ichikawa (2016; Figure 3) and scaled to our 100 ms fixed lag.

Participants were randomly assigned to one of two groups (Group 1: Aware = 24 participants and Group 2: Unaware = 28 participants). Thirty-nine out of the fifty-two participants were right hand dominant, as determined via the modified Edinburgh Handedness Inventory (Oldfield, 1971). All participants reported having no history of neurological, sensory, or motor impairment, and all had normal or corrected-to-normal vision. Prior to testing, the Ollen Musical Sophistication Index (OMSI; Ollen, 2006) was administered to establish musical sophistication, as research has demonstrated that trained musicians perform better in time discrimination tasks and may thus skew the results (Krause et al., 2010). Furthermore, video game experience was assessed as participants with considerable video game experience have been shown to perform better at TOJ and SJ tasks than those who do not have video game experience (Donohue et al., 2010). Video game experience was assessed by having participants report how many hours a week they play video games, the amount of experience they have playing games, as well as their level of expertise within different genres over the past 6 months, as done by Donohue et al. (2010).

Testing took place across two consecutive days, separated by approximately 24 h, with each testing session lasting approximately 1.5 h. The 2 days of testing differed with respect to the fixed lag (0 or 100 ms) presented between the participant’s keypress and the visual flash^1^. A fixed lag of 100 ms was used for our delayed sensory feedback as pilot data revealed that participants did not attain awareness of this lag on their own accord (see also Van Vugt and Tillmann, 2014 who reported a threshold of ∼170 ms for awareness when an auditory stimulus was used). At the end of the experiment, participants in the Unaware group completed a questionnaire that probed whether they became aware of the temporal lag (see below). Four participants in the Unaware group were classified as “aware” of the temporal lag and their data was excluded from analyses. Thus, of the 52 participants, the data from 48 participants were included in our final analyses and results reported below.

### Stimuli and Apparatus

**FIGURE 1:**
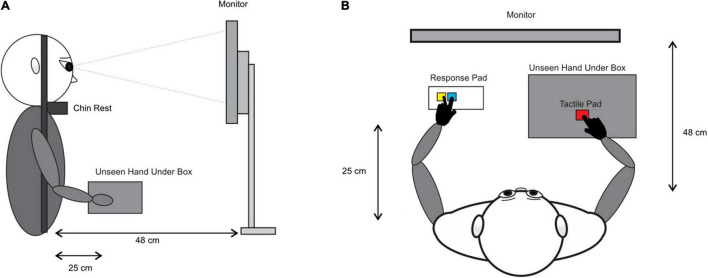
Experimental apparatus. **(A)** Side view of experimental setup with occluded vision of the hands. **(B)** Overhead view of experimental setup.

Experimental testing took place in a dimly lit room. Participants were seated in a comfortable position with their chin on a chinrest that was positioned 48 cm from a 24-inch LCD monitor (ASUS, refresh rate of 144 Hz; see Figure 1). Participants were instructed to place the index finger of their dominant hand on the tactile pad (AT42QT1010 microchip), which was covered by a box to prevent vision of the limb (see Figure 1A). The index and middle fingers of their non-dominant hand were placed on yellow and blue response keys, respectively, to complete the perceptual judgment tasks (see Figure 1B). In the perceptual judgment tasks, the index finger on the yellow key was used to indicate “FLASH AFTER KEYPRESS?” (TOJ), or “NOT SIM” (for not simultaneous in the SJ), while the middle finger on the blue key was used to indicate “FLASH BEFORE KEYPRESS? (TOJ), or “SIM” (for simultaneous in the SJ).

Visual stimuli were created using Java (version 8) and stimuli were presented on a black background on all trials. In the single tap blocks, visual stimuli included a white fixation cross (2 × 2 cm) presented at the center of the display which served as the go signal and a white or pink square (2 × 2 cm) that flashed 0.5 cm above it. Participants judged the timing of this flash relative to their keypress. In the repetitive tap blocks, a yellow or pink square (2 × 2 cm) served as the pacing stimulus in the keypress training and adaptation blocks. In the practice and test blocks, participants judged the timing of a white square (2 × 2 cm) that flashed for 50 ms.

### Procedures

**FIGURE 2:**
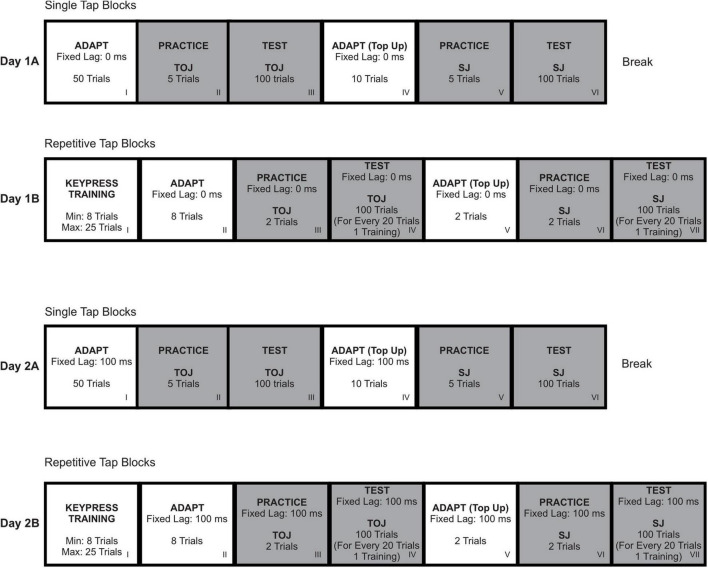
The order in which participants completed the differing fixed time lags, motor tasks (Single Tap vs. Repetitive Tap), and perceptual judgment tasks (TOJ vs. SJ) was counterbalanced across participants.

The following procedure is described for the 0 ms fixed lag trials and one specific order of motor and perceptual task conditions only. All participants completed all blocks of trials as shown in Figure 2. Trials varied with respect to the fixed temporal lag (0 or 100 ms) between keypress and visual flash, the motor task (single or repetitive tap), and perceptual judgment task (TOJ or SJ). Participants completed all blocks with a fixed temporal lag (0 or 100 ms) on the same testing day. The order of the motor tasks (single or repetitive tap) and judgment tasks (TOJ or SJ) were then counterbalanced within each testing day with some restrictions. Specifically, all blocks related to a particular motor task (single or repetitive task) were completed before starting on the second motor task. In addition, the order in which the perceptual judgment tasks were completed was the same across motor tasks.

#### Single Tap Adaptation Block

**FIGURE 3:**
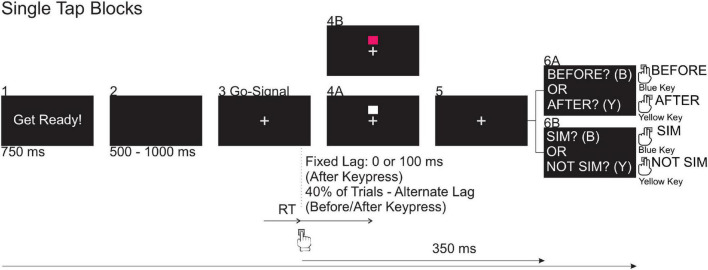
*Blocks 1–5*: Visual events occurring during the (50) adaptation trials in the single tap blocks. In the single tap practice and test blocks, participants completed *Blocks 1–6*. During these trials, the white square flashed at a fixed lag (0 or 100 ms) on 60% of trials. For the remaining 40% of trials, the white square flashed at one of 10 alternate lags relative to the expected keypress reaction time: ±15, 45, 75, 105, and 135 ms, where “+” corresponds to a lag presentation time following the expected keypress and “–” corresponds to a lag presentation time before the expected keypress.

The single tap adaptation block (Figure 2, Row 1, Block I, Day 1A) consisted of 50 trials. The timeline of trial events is shown in Figure 3. At the beginning of a trial, the words “Get Ready!” appeared on the display for 750 ms followed by a blank screen for 500–1,000 ms. A fixation cross then appeared for the duration of the trial and served as the go signal. Participants were instructed to press the tactile pad with the index finger of their dominant hand as quickly as possible when the go signal appeared. Immediately after their keypress, a square flashed above the go signal (lag of 0 ms) for 50 ms. Following the offset of the flash, feedback regarding reaction time (RT) was provided. More specifically, if the RT was <100 ms, the experimenter indicated the response was “too fast.” If the RT was >1,000 ms, the experimenter indicated that the response was “too slow.” These trials were discarded and repeated at the end of the block of trials. After RT feedback was provided, participants reported the colour of the square (white or pink, which was displayed on 38 and 12 trials, respectively). The purpose of this secondary task was to ensure that participants were paying attention to the presentation of the flash. The next trial was initiated after a participant’s response was recorded by the experimenter.

#### Single Tap Practice and Test Blocks

Following the single tap adaptation block, participants completed the practice TOJ (Figure 2, Row 1, Block II, Day 1A) and test TOJ (Figure 2, Row 1, Block III, Day 1A) blocks. These trials were similar to the single tap adaptation block except that, after each trial, participants completed a perceptual judgment task about the timing of the appearance of the visual square in relation to their keypress. Participants completed five trials in the practice TOJ block. In three of these five trials, the flash was presented approximately 200 ms *before* their keypress. In the other two trials, the flash was presented 200 ms *after* their keypress. In the test TOJ block, participants completed 100 trials in which the flash had the same fixed lag (0 ms) as in the adaptation block on 60% of the trials. In the remaining 40% of trials, the flash was randomly presented at one of ten alternate lags (i.e., SOA = ±15, 45, 75, 105, and 135 ms) with equal probability. A positive (+) SOA indicates that the flash appeared after the keypress and a negative (−) SOA indicates that the flash appeared before the expected keypress. The time of the expected keypress was predicted based on a participant’s average RT on the previous five trials. Similar to the trials in the adaptation block, the flash was presented for 50 ms. Three hundred and fifty ms after the keypress, “FLASH BEFORE KEYPRESS? OR FLASH AFTER KEYPRESS?” was displayed on the screen and participants made an unspeeded TOJ with their non-dominant hand, pressing the blue key with their index finger if they perceived the flash to come first, or the yellow key with their middle finger if they perceived their keypress to have come first (Figure 3, Box 6A).

Following the test TOJ block, participants completed a single tap adaptation “top-up” block of 10 trials with a fixed lag of 0 ms (Figure 2, Row 1, Block IV, Day 1A). These trials were identical to those in the single tap adaptation block, but only white squares were flashed. Next, participants completed the practice SJ (Figure 2, Row 1, Block V, Day 1A) and test SJ (Figure 2, Row 1, Block VI, Day 1A) blocks. The SJ trials differed from the TOJ trials with respect to the perceptual judgment task performed. The message on the screen displayed “SIM? OR NOT SIM?” and participants were instructed to report if their keypress was simultaneous with the flash or not. They used their non-dominant hand and pressed the blue key with their index finger if they perceived their keypress to be simultaneous with the flash or the yellow key with their middle finger if they perceived the two events were not simultaneous (Figure 3, Box 6B). Participants then took a break for at least 5 mins before starting the repetitive tap blocks (Figure 2, Row 2, Day 1B).

#### Repetitive Tap Keypress Training Block

In the repetitive tap blocks (Figure 2, Row 2, Blocks I–VII, Day 1B), participants first completed a keypress training block (Figure 2, Row 2, Block I, Day 1B) to learn to tap at the required pace [i.e., an inter-tap interval (ITI) of 650 ms; acceptable range = 500–800 ms]. This training block was divided into two. On the first half of the trials, the words “Get Ready!” appeared on the display. Participants tapped six times on the tactile pad with the index finger of their dominant hand in tempo with a yellow square that appeared in the centre of the display above the fixation cross (ITI of 650 ms). To determine if participants were able to match the desired tapping pace, only the ITI between taps 2–6 were analysed. If participants tapped too quickly (i.e., ITI < 500 ms), the experimenter said, “Too fast.” If they tapped too slowly (i.e., ITI > 800 ms), the experimenter said, “Too slow.” If one or more taps were too fast, and one or more taps were too slow, the experimenter said, “Out of range.” In order to move on to the second half of the training block, participants had to tap at the required pace on eight out of twenty-five trials (Figure 2, Row 2, Block I, Day 1B). If a participant could not meet the pacing demands, they were asked to withdraw from the experiment. The second half of the training block was similar to the first half; however, the yellow squares were not presented to assess the participants’ ability to tap at the required pace without an external source (i.e., the yellow square). Again, participants had to tap at the required pace on eight out of twenty-five trials to continue in the experiment. All participants were able to meet the required tapping pace in both blocks of trials.

#### Repetitive Tap Adaptation Block

**FIGURE 4:**
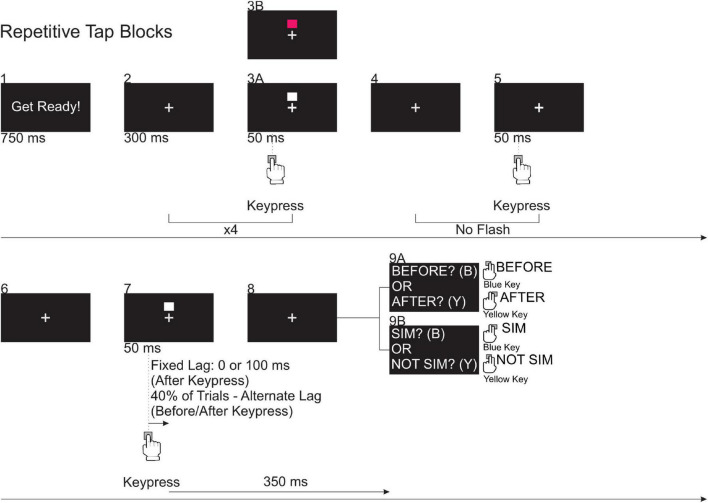
*Blocks 1–5*: Visual events occurring during the (8) trials in the repetitive tap adaptation block. In the repetitive tap practice and test blocks, participants completed *Blocks 1–9*. During these trials, the white square flashed at a fixed lag (0 or 100 ms) following the 6th keypress on 60% of trials. For the remaining 40% of trials, the white square flashed at one of 10 alternate lags relative to the expected time of the final keypress: ±15, 45, 75, 105, and 135 ms, where “+” corresponds to a lag presentation time following the expected keypress and “–” corresponds to a lag presentation time before the expected keypress.

The repetitive tap adaptation block (Figure 2, Row 2, Block II, Day 1B) followed the keypress training block and consisted of eight trials in which participants completed six taps per trial. Participants were instructed to tap at the same pace as they did in the keypress training block. The trials in the repetitive tap adaptation block included squares that flashed for 50 ms after a keypress at a fixed lag of 0 ms. On the majority of taps in a trial, the squares were white but on a small subset (1 or 2 taps per trial), the square was pink. Of the eight trials in the repetitive tap adaptation block, four trials had two pink squares within them and on the other four trials, there was only one pink square (Figure 4, Box 3B). Following each trial, participants received feedback from the experimenter regarding the timing of their taps relative to the required pace, as in the training trials (Figure 2, Row 2, Block I, Day 1B). Participants were also required to verbally report to the experimenter the number of times a pink square was presented within a trial. Similar to the single tap adaptation block, the purpose of this secondary task was to ensure that participants were paying attention to the presentation of the flash.

#### Repetitive Tap Practice and Test Blocks

After completing the repetitive tap adaptation block, participants completed the practice TOJ (Figure 2, Row 2, Block III, Day 1B) and test TOJ (Figure 2, Row 2, Block IV, Day 1B) blocks. Similar to the repetitive tap adaptation block, a white square flashed with a fixed lag of 0 ms after the keypress on the first four taps. However, no square was flashed with the fifth tap to prevent the use of timing intervals between feedback flashes as a cue for the perceptual judgment task (Figure 4, Box 5) (Tsujita and Ichikawa, 2016). A square flashed with the sixth tap. In the practice TOJ block (Figure 2, Row 2, Block III, Day 1B), there was one trial in which the flash occurred 200 ms *before* the expected sixth keypress, and on trial in which the flash occurred 200 ms *after* the sixth keypress. The test TOJ block (Figure 2, Row 2, Block IV, Day 1B) consisted of 100 trials in which the flash had the same fixed lag (0 ms) as in the adaptation block on 60% of the trials. In the remaining 40% of trials, the square was randomly flashed at the same SOAs used in the single tap task before or after the expected sixth keypress. The expected time of the sixth keypress was estimated within each trial by averaging the ITIs between taps 2 and 5.

Following the sixth keypress, the message, “FLASH BEFORE KEYPRESS? OR FLASH AFTER KEYPRESS?” was displayed. Participants made an unspeeded judgment regarding the timing of the flash by pressing the appropriate key on the response pad (see Figure 4, Box 9A). After every 20 trials in the test TOJ block, participants completed two training trials (one with the pacing stimuli (white squares) and one without; Figure 2, Row 2, Block I, Day 1B). Participants’ tapping pace was monitored and feedback was provided after both trials.

Following the test TOJ block, participants completed a repetitive tap “top-up” adaptation block which consisted of two trials (Figure 2, Row 2, Block V, Day 1B). Each trial required the completion of six taps and participants were told to tap with the same pacing that they had learned earlier in the keypress training block. The squares flashed for 50 ms following all six keypresses at a fixed lag of 0 ms.

Next, participants completed the practice SJ (Figure 2, Row 2, Block VI, Day 1B) and the test SJ (Figure 2, Row 2, Block VII) blocks. The practice SJ and the test SJ blocks were similar to those in the practice TOJ and test TOJ blocks; however, participants were now asked to judge if the events were simultaneous (“SIM?”) or not simultaneous (“NOT SIM?”) (Figure 4, Box 9B).

### Awareness Details and Probe of Awareness

Specific information regarding the timing between the keypress and flash were only provided to participants in the Aware group. These Aware participants were told about the relationship between their keypress and subsequent flash before the adaptation blocks in the experiment. For example, in the adaptation block with a fixed lag of 0 ms, they were informed that, “The flash will appear immediately after your keypress with no lag.” In the adaptation block with a fixed lag of 100 ms they were told that, “The flash will appear at a slight lag, specifically 100 ms, after you press the key”. In contrast, participants in the Unaware group did not receive any information about the relationship between their keypress and subsequent flash.

At the end of the experiment on Day 2 the Unaware group was probed on their awareness of the relationship between their keypress and flash. At that time, they were asked if they noticed whether anything had changed between Day 1 and Day 2 of the experiment with respect to the stimuli. If they answered yes, they were asked, “What do you think was going on?” If they did not mention anything about the timing interval between their keypresses and flashes, they were prompted with the following question, “Were you aware there was a lag between your keypress and resulting flash on one of the testing days?” If participants indicated, “Yes,” they were asked, “Which day was the lag presented on?” To be classified as “aware,” participants needed to specifically mention that the timing interval between their keypress and flash changed between testing days. Furthermore, they had to correctly indicate on which testing day (1 or 2), the lag was presented. Otherwise, they were classified as “unaware.”

## Statistical Analysis

### Participant Screening

In accordance with Ollen (2006), participants who scored <500 were classified as “less musically sophisticated,” while those who scored >500 were classified as “more musically sophisticated.”

Participants were classified as a “video game player” if they met the criteria put forth by Donohue et al. (2010). In particular, participants who played first-person shooter games for at least 2 h a week, or any type of action game (e.g., first-person shooter, real-time strategy, and sports games) for a minimum of 4.5 h a week and had played first-person shooter games for at least 5 h per week at some point in their lives were classified as a “video game player.” We classified participants who did not meet the preceding criteria as a “non-video game player.”

### Dual-Task Performance (Adaptation Blocks)

To ensure that participants were paying attention during the adaptation blocks (Figure 2, Row 1, Block I; Row 2, Block II; Row 3, Block I; Row 4, Block II), the percentage of correct responses regarding the colour of the square (single tap task) and the number of pink squares (repetitive tap task) were calculated for each participant. A participant’s data was only included in the subsequent analyses if they achieved an overall score of ≥90% in the adaptation blocks for both motor tasks (single tap and repetitive tap).

### Temporal Order Judgment Task

The alternate lags in which the flash preceded the keypress reflect the intended SOA and not the actual timing of the flash presentation, given the potential variation between the predicted time of keypress and actual time that participants pressed the key. Similar to the approach taken by Tsujita and Ichikawa (2016), we binned the actual lags associated with flash presentation into 30 ms intervals from −300 to 300 ms and determined the probability that a participant reported “flash after keypress” at the median temporal lag for each interval. A sigmoid function (Equation 1; Basharat et al., 2018) was fit to each participant’s responses (i.e., “flash after keypress”) across all SOAs for each TOJ testing block using Sigmaplot version 14.5. The equation used was as follows:


(1) $$f\left(x\right)=\frac{1}{\left(1+e^{\left(\frac{-\left(x-x\emptyset \right)}{b}\right)}\right)}$$

where *xø* is the PSS^2^ and reflects the SOA at which a participant indicated that their keypress occurred after (or before) the flash 50% of the time. The value of parameter b represents roughly half the range between SOAs at 25 and 75% and was used as a proxy for the temporal window of integration (TWI) as done by Basharat et al. (2018; see also Sugano et al., 2010), in which the TWI represents the discrimination sensitivity.

### Simultaneity Judgment Task

Similar to the TOJ tasks, the actual time of flash presentation relative to the keypress was first determined. The probability that a participant reported “simultaneous” was then determined at the median temporal lag for each 30 ms interval between −300 and 300 ms. A Gaussian function (Equation 2; Basharat et al., 2018) was then fit to each participant’s responses (i.e., “simultaneous”) across all SOAs for each SJ testing block using Sigmaplot version 14.5. The equation used was as follows:


(2) $$f\left(x\right)=a\times e^{\left(-0.5{\left(\frac{x-x\emptyset }{b}\right)}^{2}\right)}$$

where *a* is the amplitude fixed to 1, *xø* is the PSS, and *b* is the standard deviation (SD). The PSS (*xø*) corresponds to the SOA at the peak of the curve at which participants judged “simultaneous” the majority of the time (Harris et al., 2010). As mentioned above, the parameter *b* was used as a proxy for the TWI, in which the TWI represents the discrimination sensitivity.

The PSS and TWI were established for each participant for each testing block. Values corresponding to data with a poor fitting function (*r*^2^ < 0.5) were replaced using the multiple imputation strategy in SPSS version 27. In total, 55 PSS (∼14.3%) and 56 TWI (∼14.6%) values were replaced [see Love et al. (2013) for a similar exclusion procedure].

### Analysis

The PSS values were analysed using a 2 Group (Aware, Unaware) × 2 Fixed Lag (0, 100 ms) × 2 Motor Task (single tap, repetitive tap) × 2 Perceptual Judgment Task (TOJ, SJ) mixed analysis of variance (ANOVA) with repeated measures (RM) on the last three factors. Furthermore, for both the TOJ and SJ Perceptual Judgment Tasks, we ran a 2 Group (Aware, Unaware) × 2 Fixed Lag (0, 100 ms) × 2 Motor Task (single tap, repetitive tap) mixed ANOVA with RM on the last two factors for the TWI values. The significance value for all statistical tests was set at *p* < 0.05, and Bonferroni post-hoc tests corrected for multiple comparisons were used to find the locus of significant interactions for all pre-planned comparisons. Finally, to confirm that TR did not vary across Groups we performed Bayesian mixed ANOVAs with the factors 2 Group (Aware, Unaware) × 2 Fixed Lag (0, 100 ms) for our PSS and TWI values using standard priors as implemented by JASP version 0.15.

## Results

### Participant Screening

Only one participant in the Unaware group was deemed to be musically sophisticated (i.e., scored >500 on the OMSI). Furthermore, seven participants were identified as “video game players” (two in the Aware group and five in the Unaware group) based on our assessment of video game experience (see Donohue et al., 2010). Given that our results did not vary regardless of inclusion/exclusion of these participants, the results reported below are based on data from all 48 participants, including those deemed to be musically sophisticated or identified as video game players.

### Dual-Task Performance (Adaptation Blocks)

All participants in both the Aware and Unaware groups were able to correctly identify the square colour (single tap task) and the number of pink squares (repetitive tap task) on over 90% of trials in the adaptation blocks. In the Aware group, the mean percentage of correct responses was 99.9% (SD = 0.4) in the single tap task and 96.9% (SD = 6.7) in the repetitive tap task with a fixed lag of 0 ms and 100% (SD = 0) in the single tap task and 99.0% (SD = 3.5) in the repetitive tap task with a fixed lag of 100 ms. In the Unaware group, the mean percentage of correct responses was 99.6% (SD = 1.9) in the single tap task and 100% (SD = 0) in the repetitive tap task with a fixed lag of 0 ms, and 100% (SD = 0) in the single tap task and 97.4% (SD = 5.2) in the repetitive tap task with a fixed lag of 100 ms.

### Point of Subjective Simultaneity

**FIGURE 5:**
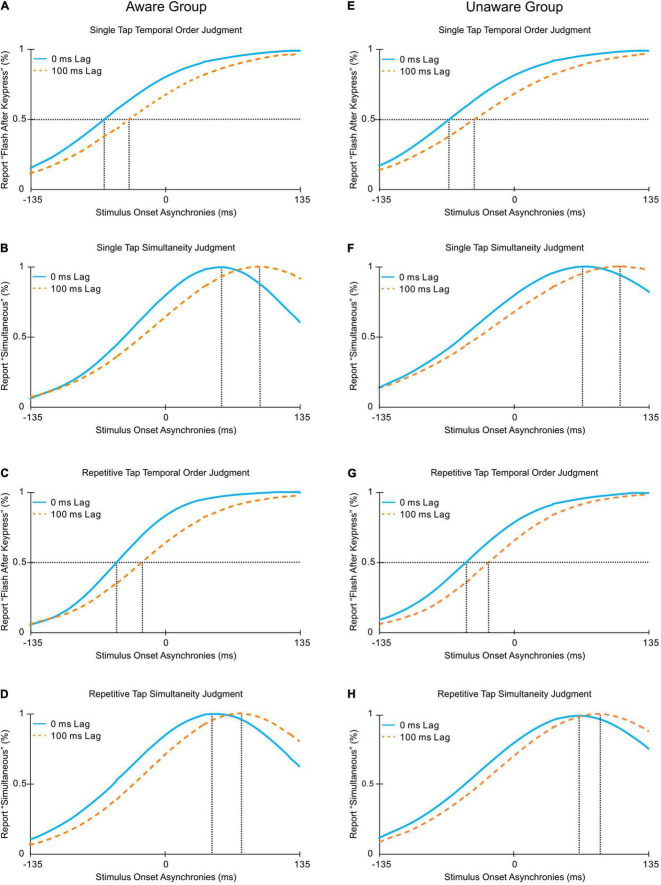
Schematic demonstrating responses in the Temporal Order Judgment (TOJ) and Simultaneity Judgment (SJ) tasks for participants in the Aware (**A–D**, left column) and Unaware (**E–H**, right column) groups in the single tap task (top two rows) and repetitive tap task (bottom two rows) in the 0 ms fixed lag block (blue) and 100 ms fixed lag block (orange). Curves reflect sigmoidal functions fit to mean participant data in the TOJ task and Gaussian functions fit to mean participant data in the SJ task across SOAs. The dashed lines in the TOJ plots indicate values at which participants responded “flash after keypress” 50% of the time (i.e., the PSS). The dashed lines in the SJ plots indicate the peak of the curves at which participants judged “simultaneous” the majority of the time (i.e., the PSS).

**FIGURE 6:**
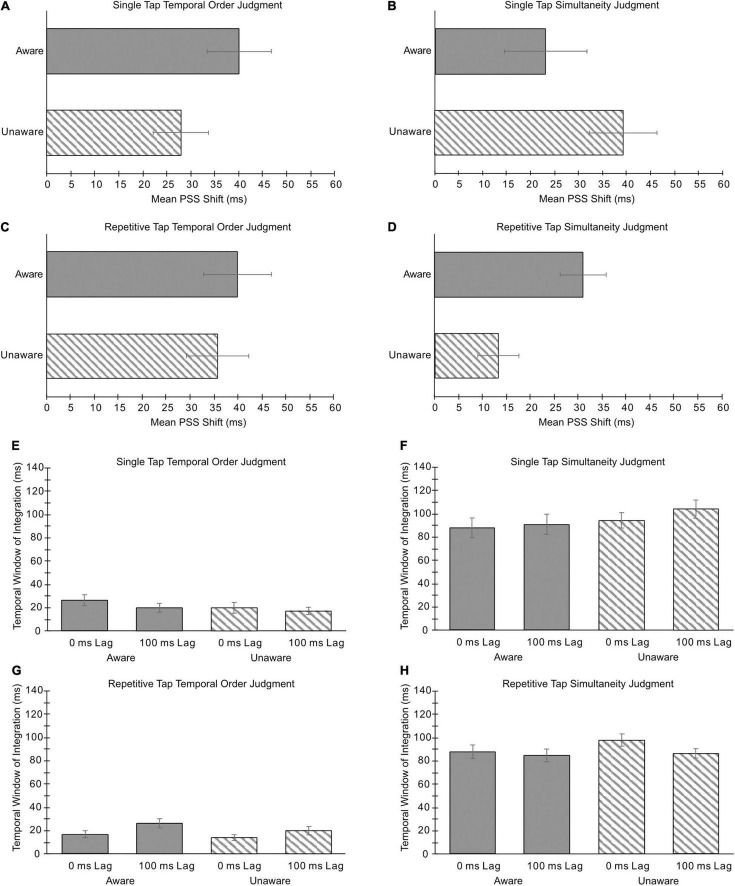
Mean shifts in the points of subjective simultaneity (PSS) (**A–D**, first two rows), and temporal windows of integration (TWI) (**E–H**, bottom two rows) are shown for the Aware (solid) and Unaware (striped) participants in the single tap task [first **(A,B)** and third **(E,F)** rows] and repetitive tap task [second **(C,D)**, and fourth **(G,H)** rows] for temporal order judgments (TOJ; left column) and simultaneity judgments (SJ; right column). Data related to the TWI are shown for the 0 and 100 ms lag blocks. Error bars denote standard error of the mean.

Functions fitted to the means (see Figures 5A–H) of each group’s responses are plotted across SOAs for blocks of trials in which the fixed lag was 0 ms (blue lines) and 100 ms (orange lines). For all motor and perceptual judgment tasks, a shift in PSS can be observed between blocks of trials with a fixed lag of 0 ms compared to 100 ms (see Figures 6A–D). These observations were supported by statistical analyses which revealed a significant main effect of Fixed Lag [*F*(1,46) = 67.226, *p* < 0.001, ηp^2^ = 0.594], with a smaller PSS value for blocks of trials in which the fixed lag was 0 ms (*M* = 19.0 ms) compared to 100 ms (*M* = 49.8 ms). The ANOVA revealed no significant main effect of Group [*F*(1,46) = 0.363, *p* = 0.550, ηp^2^ = 0.008] or interaction between Group and Fixed Lag [*F*(1,46) = 0.272, *p* = 0.604, ηp^2^ = 0.006]. Bayesian analysis with standard priors confirmed that both the Aware and Unaware groups did not differ in their extent of TR by revealing very strong support for the null hypothesis using the best model for the interaction between Group and Fixed Lag (BF_10_ = 0.055; Kelter, 2020), suggesting that the magnitude of TR observed did not differ with participant awareness (see Figures 6A–D).

While awareness did not influence the magnitude of TR observed, ANOVA revealed a significant main effect of Perceptual Judgment Task [*F*(1,46) = 443.499, *p* < 0.001, ηp^2^ = 0.906]. Post hoc analyses revealed a significant difference between the TOJ task and the SJ task in both the single and repetitive tap tasks (*p*s < 0.001). Specifically, the mean for the single tap TOJ task was −20.9 ms, while it was 88.6 ms in the SJ task. The mean for the repetitive tap TOJ task was −16.4 ms, while it was 85.3 ms in the SJ task. No other significant main effects or interactions were observed (all *p*s > 0.05).

### Temporal Window of Integration

The mean temporal window of integrations (TWIs) for the Aware and Unaware groups across all blocks of test trials are shown in Figures 6E–H. For the TOJ task, analysis revealed that the main effect of Group and Group × Fixed Lag interaction were not significant [Group: *F*(1,46) = 1.533, *p* = 0.222, ηp^2^ = 0.032: Group × Fixed Lag: *F*(1,46) = 0.035, *p* = 0.852, ηp^2^ = 0.001; BF_10_ = 0.019], with the Aware group (*M* = 22.2 ms, SD = 19.2 ms) demonstrating a similar mean TWI to the Unaware group (*M* = 18.1 ms, SD = 17.9 ms). ANOVA revealed a significant two-way interaction between Motor Task and Fixed Lag [*F*(1,46) = 5.833, *p* = 0.020, ηp^2^ = 0.113], however, *post hoc* analysis revealed that the TWI in the 0 ms Fixed Lag did not differ from the TWI in the 100 ms Fixed Lag for both the single (*p* = 0.932) and repetitive tap tasks (*p* = 0.095). For the SJ task, analysis revealed that the main effect of Group and Group × Fixed Lag interaction were not significant [Group: *F*(1,46) = 1.691, *p* = 0.200, ηp^2^ = 0.035; Group × Fixed Lag: *F*(1,46) = 0.012 *p* = 0.915, ηp^2^ = 0.000; BF_10_ = 0.057], with the Aware group (*M* = 87.7 ms, SD = 35.6 ms) demonstrating a similar mean TWI to the Unaware group (*M* = 95.9 ms, SD = 30.5 ms). No other significant main effects or interactions were observed (*p*s > 0.05).

## Discussion

The current study looked to establish whether awareness of a temporal lag between one’s keypress and visual flash is necessary for TR. Moreover, we investigated if motor and perceptual judgment task demands modulate the influence of awareness on TR. We found that participants demonstrated significant TR across all motor (single and repetitive tap) and perceptual judgment (TOJ and SJ) tasks. Contrary to our predictions, the magnitude of TR did not differ statistically between our Aware and Unaware groups across all motor and perceptual judgment tasks. Moreover, we showed that awareness did not lead to significant differences in discrimination sensitivity (i.e., TWI) between groups. That is to say, the Aware and Unaware groups both demonstrated similar discrimination sensitivity when determining which event occurred first or if they occurred simultaneously. These null finding are supported by our large sample size and Bayesian statistics. Thus, based on our results, we suggest that awareness of the temporal lag does not have an impact on TR and that task demands do not modulate the influence of awareness on TR. Furthermore, TR can be induced regardless of one’s awareness of the temporal lag between their keypress and appearance of a visual stimulus.

### Awareness and Temporal Recalibration

Recent work by Tsujita and Ichikawa (2016) suggests that awareness of a temporal lag between one’s keypress and the appearance of a visual flash plays a critical role in TR. This was based on their observation that only participants who were made aware of the temporal lag between their motor action and visual stimuli showed TR. To induce awareness, Tsujita and Ichikawa (2016) explicitly informed a subset of their participants (Aware group) of the 200 ms fixed temporal lag between their keypress and the appearance of a visual stimulus when completing a repetitive tap task in which a TOJ was required. The other subset of their participants (Unaware group) was not explicitly informed about the fixed temporal lag. Instead, this Unaware group was gradually introduced to the 200 ms fixed lag over five blocks of 20 trials, such that the lag increased by 40 ms every 20 trials until the 200 ms temporal lag was achieved. Their results indicated that only participants who were made aware of the temporal lag demonstrated significant TR. Tsujita and Ichikawa (2016) also showed that the TWI did not differ significantly between their Aware and Unaware groups.

To date, research regarding the role of awareness on TR is limited. Most studies examining TR have not directly manipulated or assessed participants’ awareness of the temporal lag (Stetson et al., 2006; Stekelenburg et al., 2011; Parsons et al., 2013; Timm et al., 2014; Vercillo et al., 2015). Gallagher et al. (2014) reported that awareness was necessary for TR, but they conceptualised awareness as the ability to perceive visual stimuli that were presented subliminally. In other work, Heron et al. (2010) examined the role of selective attention within a sensory-sensory TR paradigm. In their audio-visual paradigm, participants’ attention was directed to either the fixation cross, the stimuli, or the order in which the stimuli appeared (i.e., the temporal structure). Participants were asked to judge which stimulus occurred first. They found that when participants focused on temporal structure, there was a greater magnitude of TR than when they focused on irrelevant stimulus features. This demonstrated that TR was driven by top-down factors. However, awareness of the temporal lag was not specifically assessed and the relationship between selective attention and awareness has yet to be determined.

In the current study, we manipulated participants’ knowledge of the temporal lag by explicitly telling our Aware participants about the temporal lag between their keypress and the visual flash. The Unaware participants were not told about the temporal lag and our probe at the end of the experiment revealed that the majority of participants in our Unaware group were not aware of the fixed lag between their keypress and visual stimulus with the exception of four participants. Consequently, the data of these four participants were removed from the analyses. When the magnitude of TR and TWI were compared between our Aware and Unaware groups, no significant differences were found. Both groups demonstrated TR of approximately 30 ms, such that there was a rightward shift of 30 ms in PSS values between blocks of trials with a fixed temporal lag of 0–100 ms. These results indicate that participants perceived simultaneity 30 ms later in the block of trials with a fixed lag of 100 ms compared to 0 ms. This magnitude of TR was observed across all motor tasks (single and repetitive tap) and perceptual judgment tasks (TOJ and SJ), independent of participant awareness. Together, these results suggest that we may have manipulated conceptual awareness as opposed to perceptual awareness, as providing knowledge of the temporal lag does not mean that participants necessarily perceived the delay between their keypress and visual flash (i.e., perception of the temporal lag could have been similar between our Aware and Unaware groups).

Differences in methodology and hardware limitations may explain why our results differ from those of Tsujita and Ichikawa (2016). First, with respect to methodology, the two studies differed with respect to the manner in which the fixed temporal lag was introduced, and the magnitude of the fixed lag itself. Tsujita and Ichikawa gradually introduced their Unaware group to the 200 ms temporal lag over the span of 100 trials. Consequently, these participants only completed 20 trials with a temporal lag of 200 ms (i.e., participants experienced 80 delayed tap-flash pairs). In contrast, our Unaware group completed all adaptation and test trials with the 100 ms temporal lag (i.e., participants experienced over 456 delayed tap-flash pairs). It is possible that the Unaware group in Tsujita and Ichikawa’s (2016) study did not have enough trials to adapt to the temporal lag of 200 ms and hence demonstrated less TR than their Aware group. It is also important to note that the temporal lag of 200 ms used by Tsujita and Ichikawa (2016) was double the 100 ms temporal lag used in the current study. A temporal lag of 200 ms has been shown to lead to a greater magnitude of TR than a temporal lag of 100 ms (see Heron et al., 2009), which may have contributed to the greater TR observed in the Aware group in Tsujita and Ichikawa’s (2016) study compared to participants in our Aware group.

Increased temporal delays due to hardware limitations may have also contributed to the larger TR observed in the Aware group of Tsujita and Ichikawa (2016) compared to our Aware group. We used a sensitive custom-made tactile response pad, creating minimal delays in our system (i.e., delays between 17 and 21 ms). In contrast, participants in Tsujita and Ichikawa’s (2016) study responded using a Dell SK-8175 keyboard, which was connected to a CRT display (SONY CPD-G200J). As Sugano et al. (2010) note, the amount of time for a keypress to be read can take over ∼25 ms whereas custom-made tactile response pads can be as fast as ∼1 ms. Moreover, it is not known what event (i.e., criteria) participants used to judge when the motor task was completed. That is to say, do participants bias their judgment with respect to when they initiate their keypress or when the key is fully depressed or when the key is released? The tactile pad that participants used to make their responses in the current study only required a light touch, resulting in consistency in the time-course of trial events. Studies requiring participants to use a keyboard tend to find larger variability and/or greater magnitudes of TR (Stetson et al., 2006; Vercillo et al., 2015; Tsujita and Ichikawa, 2016) compared to studies in which participants complete their responses on a tactile pad (Sugano et al., 2010; Stekelenburg et al., 2011; Keetels and Vroomen, 2012). This difference in the magnitude of TR arising between studies employing keyboards versus tactile pads may be attributed to participants’ equivocal decision-making strategies regarding how they judged the temporal order or simultaneity of their keypress relative to the sensory stimulus. To improve consistency within and across participants regarding the criteria used to establish the timing of one’s motor response, future studies should look to use a tactile pad with limited delays. For now, we show that awareness does not appear to modulate TR when participants have ample time to adapt to a fixed temporal lag of 100 ms and when temporal delays due to hardware limitations are controlled for, i.e., use of a tactile pad instead of a keyboard.

### Motor Responses

Two motor tasks commonly used in studies examining TR are the single tap and the repetitive tap tasks (Stetson et al., 2006; Heron et al., 2009; Sugano et al., 2010; Stekelenburg et al., 2011; Keetels and Vroomen, 2012; Parsons et al., 2013; Yarrow et al., 2013; Timm et al., 2014; Vercillo et al., 2015). Across the literature, the magnitude of TR has been shown to vary in a repetitive tap task. For example, Stekelenburg et al. (2011) found a TR magnitude of 9 ms when participants adapted to a fixed lag of 100 ms, whereas Tsujita and Ichikawa (2016) found a TR magnitude of approximately 99 ms when participants adapted to a fixed lag of 200 ms. However, when a single tap task was used, the magnitude of TR was shown to be more consistent across experiments, such that a TR magnitude of approximately 40 ms has been repeatedly reported regardless of whether the fixed lag was 100 ms (see Stetson et al., 2006; Timm et al., 2014) or 200 ms (Vercillo et al., 2015). We found a TR magnitude of approximately 30 ms, which did not differ significantly between motor tasks (single or repetitive tap).

The single tap task requires participants to complete a discrete tap in response to a go signal. In contrast, the repetitive tap task is sequential, requiring participants to maintain a tapping rhythm and determine the timing of the last tap to halt the sequence. Previous reports have attributed the varying pattern of TR observed between these two motor tasks to the engagement of different cognitive processes (see Stekelenburg et al., 2011); though this assumption was based on comparisons of results across various experiments differing in methodologies (Stetson et al., 2006; Sugano et al., 2010). The current study is the first to directly compare the magnitude of TR between a single tap task and a repetitive tap task within the same experiment. In doing so, we were able to maintain consistency with respect to the experimental setup, visual stimuli displayed, the timing of events, the testing environment, and the experimenter. We found that the magnitudes of TR, as well as TWI, did not differ significantly across the single tap and repetitive tap tasks. Future work is required to establish the overlap and distinction of specific cognitive processes and patterns of neural activation underlying TR in single and repetitive tap tasks.

### Perceptual Judgments

We compared TR across two perceptual judgment tasks (TOJ and SJ) in a motor-sensory paradigm. TR was established by shifts in the PSS between blocks of trials with a 0 vs. 100 ms fixed lag. A limitation to consider when interpreting changes in participants’ judgments is the impact of response bias. In each of our testing blocks, 60% of the trials had a fixed lag of 0 or 100 ms. If participants were biased to use each response option equally, then there would have been a temporal shift in the PSS values between the 0 and 100 ms fixed lag blocks in the same direction observed in the current experiment. Thus, the TR we observed in the current study may also reflect participants’ response biases. For now, we discuss our results in light of TR, however, future research is needed to distinguish TR from response bias.

Jaśkowski’s (1991) two-stage model suggests that two timing mechanisms are needed to discriminate temporal event order in a TOJ task, but only one timing mechanism needed to establish simultaneity in a SJ task. The first timing mechanism determines if the two stimuli appeared simultaneously, or not (TOJ and SJ tasks). If it is determined that the two did not appear simultaneously, a second timing mechanism decides which event occurred first (TOJ task). Thus, based on this two-stage model, the TOJ task requires additional cognitive processes compared to the SJ task. Behavioural and neurophysiological evidence supports this conclusion, as Love et al. (2013) found that participants perceived the TOJ task to be more difficult to complete than the SJ task and the TOJ task has been shown to engage additional cortical areas in comparison to the SJ task (e.g., TOJ recruits the frontal, parietal, and temporo-occipital regions while recruitment in the SJ task seems to be limited to frontal and parietal areas; Binder, 2015).

We predicted that awareness would benefit TR when completing the TOJ task compared to the SJ task given the suggestion that the TOJ requires additional cognitive processes compared to the SJ (Love et al., 2013; Binder, 2015). In contrast to our predictions, we found a similar magnitude of TR across our TOJ and SJ tasks (Love et al., 2013; Binder, 2015). Interestingly, we did find differences in the PSS across the two perceptual judgment tasks. Our results and those from previous studies (Vatakis et al., 2008; Love et al., 2013; Binder, 2015; Basharat et al., 2018) suggest that the differences in response patterns between the TOJ and SJ tasks may arise because these tasks rely on different cognitive processes.

In the current study, we defined TWI as reflecting participants’ discrimination sensitivity. The calculation of TWI values differed between our TOJ and SJ tasks and thus we did not compare TWI values across perceptual judgment tasks. Furthermore, we recognise that our TWI measure within the SJ task can be dependent upon the criteria a participant adopts to determine whether their keypress and the visual flash are “simultaneous” (i.e., if they are liberal vs. conservative when making use of the “synchronous” response option). Thus, one must be careful when interpreting results related to TWI. Keeping these considerations in mind, we did not find any group differences in TWI in the TOJ and SJ tasks.

### Conclusion

We found that TR did not differ between the Aware and Unaware groups. Specifically, we found that participants demonstrated a similar magnitude of TR regardless of whether or not they knew about the temporal lag between their keypress and visual flash across different motor (single and repetitive tap) and perceptual judgment (TOJ and SJ) tasks commonly used in the TR literature. Furthermore, we showed that the average PSS differed between the TOJ task and the SJ task, which suggests that these perceptual judgment tasks are likely subserved by different cognitive processes. Since our results are purely behavioural, future studies should look to identify the neural activation underlying TR.

## Data Availability Statement

The original contributions presented in the study are publicly available. This data can be found here: https://doi.org/10.7910/DVN/J6KTRS.

## Ethics Statement

The studies involving human participants were reviewed and approved by University of Ottawa’s Health Sciences and Science Research Ethics Board. The patients/participants provided their written informed consent to participate in this study.

## Author Contributions

MB contributed to the conception, design, acquisition, data analysis, interpretation of data, drafting, and critically revising the work for intellectual content. ML contributed to critically revising the work for intellectual content. EC contributed to the funding, conception, design, critically revising the work for important intellectual content, provided approval for publication, and agreed to be accountable for all aspects of the work in ensuring that questions related to the accuracy or integrity of any part of the work are appropriately investigated and resolved. All authors contributed to the article and approved the submitted version.

## Conflict of Interest

The authors declare that the research was conducted in the absence of any commercial or financial relationships that could be construed as a potential conflict of interest.

## Publisher’s Note

All claims expressed in this article are solely those of the authors and do not necessarily represent those of their affiliated organizations, or those of the publisher, the editors and the reviewers. Any product that may be evaluated in this article, or claim that may be made by its manufacturer, is not guaranteed or endorsed by the publisher.

## Footnotes

1 We recognise that on all trials there was an inevitable lag between one’s keypress and the appearance of the visual flash due to hardware limitations. Previous studies (e.g., Stetson et al., 2006; Tsujita and Ichikawa, 2016) have reported average delays between 35 and 59 ms and did not include these hardware limitations when reporting the inserted lag between keypress and visual flash (i.e., reported a fixed lag of 0 or 100 ms as we have done). The delay between the tactile pad and the PC in our setup was between 17 and 21 ms (verified through Python and Arduino) and the delay between the PC and the monitor was 3 ms. We did not include this delay when reporting the inserted lag between the keypress and visual flash (see Stetson et al., 2006).

2 The sigmoid and Gaussian function curves were fitted to the data using the Levenberg–Marquardt algorithm (i.e., dampened-least squares method) as done by Basharat et al. (2018).
